# Transient urocolpos due to vesicovaginal reflux in a 37-year-old woman: a case report

**DOI:** 10.11604/pamj.2022.43.37.36668

**Published:** 2022-09-21

**Authors:** Muneer Fazea, Ramzi Alnajmani, Mansour Alhameli, Faisal Ahmed, Abdullah Al-Naggar, Abdulfattah Altam, Waleed Aljbri, Qasem Alyhari, Amal Bafagih, Mohamed Badheeb

**Affiliations:** 1 Department of Radiology, School of Medicine, Ibb University of Medical Sciences, Ibb, Yemen,; 2 Department of Radiology, Al-Maamoon Diagnostic Center, Sana´a, Yemen,; 3 Department of Radiology, School of Medicine, 21 September University, Sana'a, Yemen,; 4 Department of Radiology, Faculty of Medicines, Sana´a University of Medical Science, Sana´a, Yemen,; 5 Urology Research Center, Al-Thora General Hospital, Department of Urology, School of Medicine, Ibb University of Medical Sciences, Ibb, Yemen,; 6 Department of Anesthesiology, Al-Thora Modern Hospital, Faculty of Medicine, Sana´a University of Medical Sciences, Sana´a, Yemen,; 7 Department of General Surgery, School of Medicine, 21 September University, Sana'a, Yemen,; 8 Department of Urology, School of Medicine, 21 September University, Sana'a, Yemen,; 9 Department of General Surgery, School of Medicine, Ibb University of Medical Sciences, Ibb, Yemen,; 10 Department of Internal Medicine, Faculty of Medicine, Hadhramaut University, Hadhramaut, Yemen

**Keywords:** Urocolpos, incontinence, vesicovaginal reflux, computed tomography scan, case report

## Abstract

Urocolpos is a urine collection in the vagina, possibly due to vesicovaginal reflux (VVR) or a fistula. Urocolpos is a rare phenomenon in adults, and it is commonly underrecognized by radiologists. The occurrence of urocolpos due to VVR after a Caesarean section in adult females is a rare phenomenon; failure to recognize it may lead to misdiagnosis as vesicovaginal fistula and often leads to unnecessary medical interventions. We report a 37-year-old female who complained of abdominal pain and intermittent daytime urinary incontinence for six months, starting after a Caesarean section for her third baby. An abdominal computed tomography scan revealed a large fluid-filled vagina on a full bladder without anatomical abnormality. A post-voiding scan showed a complete resolution. In conclusion, urocolpos is an underrecognized entity that rarely coexists with VVR. Post-voiding resolution and vaginal contrast reflux are important findings that can aid radiologists in identifying such cases.

## Introduction

Urocolpos is a urine collection in the vagina, possibly due to vesicovaginal reflux (VVR) or a fistula in adults [1]. It is commonly seen in children and is caused by an anatomical abnormality such as the vaginal septum, hypoplastic vagina, or imperforate hymen [2]. VVR typically occurs in young children and prebuttal females with a prevalence rate of 12-15% and is anticipated to become nearly absent in middle age [3]. This condition has a variable clinical presentation, including abdominal pain, abdominal distension, urinary incontinence, recurrent urinary tract infections (UTIs), nocturnal enuresis, vaginitis, and vaginal discharge [4].

The occurrence of hydrocolpos due to VVR after a Caesarean section in adult females is very rare and may be misdiagnosed as a vesicovaginal fistula [3,5]. VVR is frequently diagnosed by urologists; however, it is less frequently diagnosed by radiologists, resulting in diagnostic challenges [2]. We present a case of intermittent daytime urinary incontinence in a 37-year-old female patient; further evaluation with computed tomography (CT) scan confirmed a VVR diagnosis.

## Patient and observation

**Patient information:** a 37-year-old married female presented with a complaint of abdominal pain for six months. The patient had a history of intermittent daytime urinary incontinence immediately after voiding with no specific relation to increased intrabdominal pressure (during coughing, straining, or laughing), urgency, and UTI that started after the Caesarean section for her third baby. She denied any history of dysuria, vaginal discharge, or hematuria. The menstrual history was unremarkable, without a history of any chronic medical condition or malignancy. The patient denied any similar problem in a previous Caesarean section.

**Clinical findings:** the patient was overweight (weight: 80 kg and height: 1.65 m, Body mass index (BMI): 29.3 kg/m^2^). Examination of the abdomen and external and internal genitalia was unremarkable.

**Figure 1:**
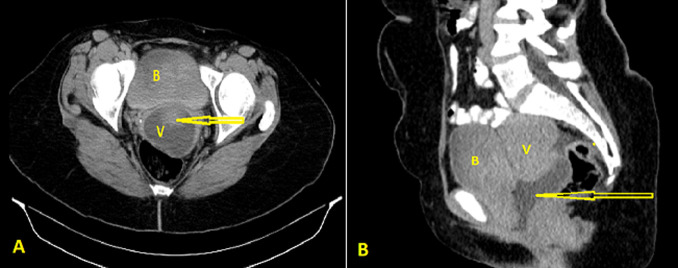
computed tomography scan showing a contrast-filled urinary bladder and vagina with no definite vesicovaginal fistula (arrow); A) axial view; B) sagittal view; (B: bladder, V: vagina)

**Figure 2:**
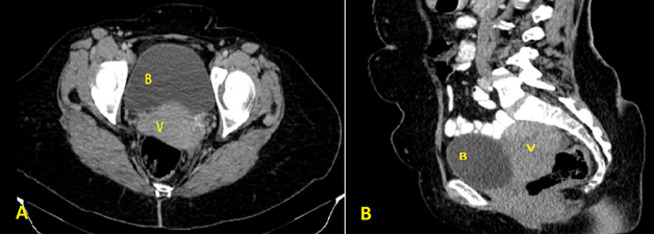
post-void computed tomography scan showing emptying of the urinary bladder with complete disappearance of the cystic lesion; A) axial view; B) sagittal view; (B: bladder, V: vagina)

**Diagnostic assessment:** blood counts and renal function tests were within normal limits. Urinalysis revealed only a few pus cells (10 WBCs/HPF). Urine culture revealed Escherichia coli sensitive to ciprofloxacin, levofloxacin, and cefixime. Transabdominal ultrasonography (USS) revealed an anechoic mass-like lesion located posterior to the urinary bladder with an associated anterior displacement and posterior acoustic enhancement. These findings were suggestive of a fluid-filled vagina (urocolpos). The adjacent abdominal structures were of normal appearance. The mass was completely unvisualized in the post-void USS image, implying a VVR diagnosis. Further evaluation with an abdominopelvic CT scan revealed a distended fluid-filled vagina on full bladder imaging suggestive of urocolpos (Figure 1). In addition, compressive effects were noted on the nearby structures with an anteriorly-displaced urinary bladder and uterus. The complete resolution of these findings in post-void CT images was confirmatory for VVR-related non-obstructive urocolpos diagnosis (Figure 2). There was no evidence of any other associated structural abnormalities, including; ectopic ureter, vesicovaginal fistula, or bladder diverticula.

**Therapeutic interventions:** the patient has been treated with levofloxacin 500 mg daily for five days. Additionally, as part of her treatment, the patient was recommended to perform behavioral therapy with a voiding retraining program, including the proper toileting position.

**Follow-up and outcome:** within nine months of follow-up, the patient´s condition was improved without recurrence of symptoms.

**Patient perspective:** during treatment, the patient was satisfied with the diagnosis and the level of care provided to her.

**Informed consent:** it was obtained from the patient for participation in our study.

## Discussion

Urocolpos is a urine accumulation in vagina that causes cystic dilatation and distension. Obstructive vaginal lesions and urogenital malformations are frequently associated with this condition. Obstructive lesions appear early in life, but VVR typically occurs in prepubertal girls with a prevalence rate of 12-15% and is anticipated to become nearly absent in middle age, which is rationally described by gradual bladder descent and anatomical modification of the vesicourethral angle [3,6,7]. There are few reports of VVR causing urocolpos in adult women, such as Motavasseli *et al*. who reported 16 (20%) cases, with a mean age of 47 years, Haouas *et al*. and Prashant *et al*. [3,8,9]. Similarly, we reported a 37-year-old adult woman with VVR. The exact pathogenesis of this condition is unknown. However, several predisposing conditions have been proposed, including labia minora adhesions, ureteral duplication or ectopic ureter with insertion into the vagina, and female hypospadias (abnormal position of the urethral meatus in relation to the vaginal orifice and labia). Other risk factors include improper toilet training (voiding with tightly opposed legs) and spastic disorders such as cerebral palsy, pelvic floor dysfunction, and obesity [3,10]. Our patient was obese and may have pelvic floor dysfunction.

The probable mechanism of pelvic floor dysfunction among obese women is due to the effect of hypoestrogenism. Additionally, obesity is associated with increased intra-abdominal pressure that may lead to pelvic floor muscle weakness [11]. These patients' symptoms were nonspecific and may present with varied clinical presentations such as UTI, abdominal pain, vaginal discharge, bedwetting even during the day, and even post-void dribbling, which is then confused with urinary incontinence, as seen in our patient. A passage of urine through the vagina after the patient has naturally voided through the urethra is one specific point that can be elicited [3].

The most common diagnostic radiologic modalities for VVR are the USS, CT scan, pelvic magnetic resonance imaging (MRI), and voiding cystourethrography (VCUG) [10]. In the absence of other structural abnormalities, the complete resolution of fluid collection in post-voiding pelvic USS is essential in diagnosing VVR with non-obstructive urocolpos. CT scan provides better visualization and can be helpful in confirming the diagnosis and ruling out the presence of an associated fistula or other urogenital tract abnormalities. MRI is superior to CT given its ability to identify the vaginal septum and differentiate hematocolpos from urocolpos, in addition to lacking radiation effect, making it a safer and more accurate utility [3,10]. VVR can be evaluated clinically by inserting an intravaginal tampon after urinary bladder emptying, and the disappearance of leakage can indicate a VVR diagnosis [3]. Another recommended test is cystoscopy which could exclude the presence of a fistula [3]. In our patient, the CT scan was enough to confirm the diagnosis.

Obstructive urocolpos and vesicovaginal fistula are associated with anatomical abnormalities that require surgical intervention. Therefore, it should be differentiated from non-obstructive urocolpos that are managed typically through behavioral therapy [8,10]. Various therapeutic approaches are used in VVR treatment, including postmicturition vaginal draining; this can be performed through intravaginal tampon placement or intravaginal finger introduction in a cross-legged position. Despite the reported improvement with these interventions, most patients are inconsistent or uncomfortable using such techniques; therefore, it´s extremely challenging to assess their effectiveness. Patients are also recommended to strengthen pelvic floor muscles with exercises that can improve the vaginal tone and reduce the wide vaginal opening [2,3]. Our patient was treated with appropriate voiding instructions (spreading the legs during voiding) and antibiotics therapy for UTIs.

## Conclusion

Urocolpos is an underrecognized entity that rarely coexists with VVR. Post-voiding resolution and vaginal contrast reflux are important findings that can aid the radiologist in identifying such cases to limit unnecessary medical and radiologic investigations, which provoke patient anxiety.
